# Mechanically-foldable axial flow blood pump: response-surface-based structural optimization and hemolytic performance evaluation

**DOI:** 10.3389/fphys.2025.1632333

**Published:** 2025-12-18

**Authors:** Tairan Ji, Teng Jing, Jianan Cheng

**Affiliations:** National Research Center of Pumps, Jiangsu University, Zhenjiang, Jiangsu, China

**Keywords:** LVAD, foldable blood pump, cage filament, response surface methodology, computational hemodynamics, parametricoptimization

## Abstract

**Introduction:**

Traditional percutaneous ventricular assist devices (PVADs) face limitations due to their small implantation size, requiring higher rotational speeds to meet left ventricular support demands. However, the elevated shear stress induced by high-speed operation leads to excessive hemolysis, necessitating design improvements for better clinical applicability.

**Method:**

This study presents a foldable implantable blood pump featuring a collapsible flexible impeller and pump casing, which reduces its profile during implantation and expands to operational size at the target position, thereby allowing lower rotational speeds and improved hemolytic performance. Through numerical simulations combined with response surface methodology (RSM), we systematically analyzed the influence of various structural parameters on pump head and hemolysis index (HI), aiming at parametric optimization of the initial model for enhanced hydraulic efficiency and reduced blood trauma risk.

**Results:**

Simulation analysis of seven parameter configurations identified five key structural parameters with dominant effects on head and HI: impeller inlet angle, impeller outlet angle, diffuser inlet angle, diffuser wrap angle, and impeller-diffuser gap. Using Box-Behnken Design (BBD), we established dual-response prediction models for both head and HI, analyzed significant interaction terms, and derived an optimized configuration achieving a 6.9% increase in head pressure and 17.9% reduction in HI compared to the initial design. Additional analysis revealed that increasing tip clearance reduces hemolysis at the cost of moderate head decrease.

**Conclusion:**

We designed and optimized a foldable axial-flow blood pump for transaortic implantation. The optimized configuration demonstrates a 6.9% head improvement to 2.346 m and 17.9% HI reduction to $1.081\times 10^{-2}\%$ . Furthermore, increasing tip clearance from 0.2  mm to 0.4 mm provides additional hemolysis reduction without excessively compromising the pressure head.

## 1 Introduction

With the aging population and increasing prevalence of unhealthy lifestyles, cardiovascular diseases have become one of the major global health threats Mensah et al. (2023). Heart failure, as one of the primary manifestations of cardiovascular diseases, severely impacts normal physiological activities of patients. For end-stage heart failure patients, heart transplantation remains the most effective treatment, but limited donor availability has made alternatives necessary McDonagh et al. (2022). Traditional VAD implantation requires open-heart surgery, which is highly invasive and carries significant complications, making it unsuitable for acute heart failure patients (Miller and Rogers, 2018; Mehra et al., 2019; Köhne, 2020). Extracorporeal membrane oxygenation (ECMO) acts well in such situations (Han et al., 2024). Han et al. (2022) conducted a simulated and experimental analysis of three leading centrifugal pumps on the market under clinical conditions of adult ECMO support, obtaining and comparing their hemolytic performance. He et al. (2021) compared the new Breethe centrifugal blood pump with two popular pumps for ECMO by a combined computational and experimental approach, finding it capable for clinical use. However, the surgery for ECMO is overly invasive, presenting an unacceptably high risk to patients with normal pulmonary function.

In recent years, minimally invasive percutaneous VADs (PVADs) have emerged as a research hotspot de Oliveira Cardoso et al. (2022). These compact devices can be implanted through arterial puncture. However, their small size necessitates higher rotational speeds, increasing hemolysis risk Köhne (2020). Attinger-Toller et al. (2022) found that while Impella devices can effectively reduce ventricular load and improve myocardial function, clinical studies revealed complications like limb ischemia. Vandenbriele et al. (2022) reported hemolysis rates ranging from 5% to 63% in micro axial-flow pumps, which may lead to increased vascular tension, platelet activation/aggregation, and arterial thrombosis, posing serious threats to patient health. Constrained by the minimal diameter of the blood vessels during the implantation procedure,the radius of PVADs is severely constrained, which poses significant challenges in controlling the rotational speed and mitigating hemolysis.

**FIGURE 1:**
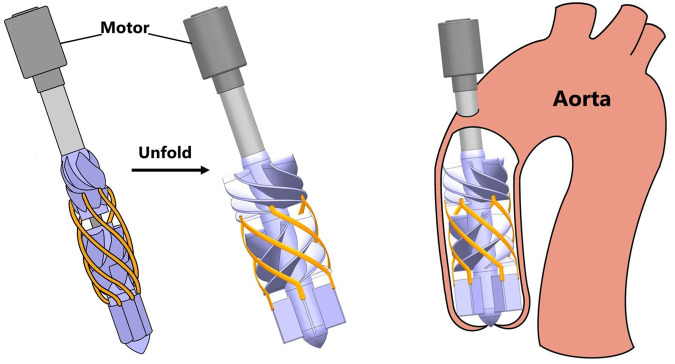
Schematic diagram of folding aortic axial blood pump.

To address these challenges, foldable aortic axial-flow blood pumps have been proposed. The foldable pump concept offers a novel paradigm for the optimization of PVADs (Pulsatile Ventricular Assist Devices). By utilizing elastic materials, these pumps can be folded during the implantation procedure and subsequently expanded upon reaching the aorta, thereby achieving a working radius unattainable by conventional PVADs. The operational principle of foldable pumps is schematically illustrated in Figure 1. This innovative approach significantly enhances the operational radius of foldable pumps, which permits a reduction in rotational speed. Consequently, this leads to lower shear stress and reduced hemolysis—-a crucial hemodynamic advantage. Given these benefits, the foldable pump design represents a promising and likely influential future direction for PVAD development.

Current research on foldable blood pumps remains limited, primarily focusing on Fontan procedure applications, which aim to increase single ventricle preload and differ functionally from LVADs for heart failure treatment. Throckmorton et al. developed a foldable propeller pump for single ventricle Fontan circulation, featuring blades hinged to the shaft that collapse within a cage filament during implantation and expand in the central vein to provide circulatory support Throckmorton et al. (2007). Hsu et al. proposed a simplified 2D analytical method for estimating stress and deformation in shape-memory alloy frames, applied to two common foldable blood pump designs Hsu et al. (2014). Hirschhorn et al. conducted one-way fluid-structure interaction analysis on a foldable axial-flow pump for Fontan patients, concluding that stiffer frame materials combined with flexible polymer coverings represented the optimal solution for foldability Hirschhorn et al. (2020). Beyond the Fontan application focus, existing research has mainly addressed material selection rather than structural optimization.

Structural optimization is one of the important parts for VAD designing, many groups have already used different opitization frameworks for VAD structural optimization. Xu et al. (2015) used neural networks and a genetic algorithm to get a best hemocompatibility for a pulsatile ventricular assist device, but they only focused on the hemocompatibility and ignored hydraulic performances. Jing et al. (2023) achived a theoratically optimal point of a screw centrifugal blood pump with randomForest and multi-objective gray wolf optimization algorithm for both hemocompatibility and hydraulic performances,but their algorithm could not find the interactions between different parameters. Bounouib et al. (2025) used RSM to optimize hemocompatibility metrics in ventricular assist device design, which could find the relationships between the parameters. Pitifully, they mainly described the influence of single parameters.

Foldable pumps employing flexible materials and cage filament structures exhibit fundamentally different mechanical properties from conventional PVADs, necessitating in-depth investigation of their structural parameters. And for these parameters, the interaction relationships between them also remains research.

This study aims to design a foldable axial-flow blood pump for transaortic implantation and identify an optimal structural configuration, and analyze the interactions between the parameters. We performed numerical simulations on the initial pump model to obtain its head and HI, conducted Plackett-Burman (PB) screening tests for key structural parameters, established dual-response prediction models for both head and HI, analyzed significant interaction terms, and ultimately derived optimized structural parameters.

## 2 Materials and methods

### 2.1 Numerical simulation of the original design

The foldable axial-flow blood pump in this study is designed for implantation in the ascending aorta. During implantation, the impeller, guide vanes, and cage filaments remain folded, expanding at the target position to achieve a larger working radius. Figure 1 illustrates the implantation process and working principle of the foldable blood pump.

**FIGURE 2:**
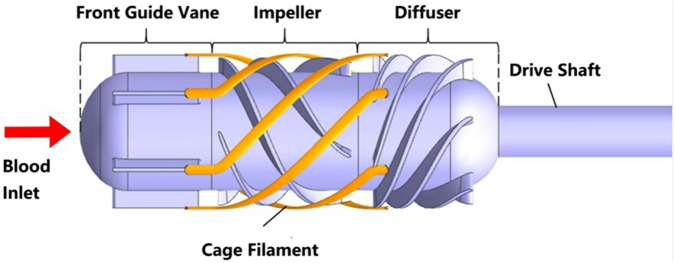
Schematic diagram of folding aortic axial blood pump.

Using blood as the working fluid, CFD simulations can predict both the hydraulic performance and erythrocyte damage caused by high-speed impeller rotation, enabling hemolysis index (HI) calculation and hemolytic performance analysis. This section details the simulation process including CFD setup, mesh generation, and hemolysis computation. Key structural parameters include: impeller blade inlet angle $\theta_{1}=17\text{°}$ , outlet angle $\theta_{2}=63\text{°}$ , wrap angle $\theta_{3}=25\text{°}$ , diffuser inlet angle $\theta_{4}=130\text{°}$ , and impeller-diffuser spacing $L_{2}=2.5\text{ mm}$ . The schematic diagram of this pump is shown in Figure 2.

#### 2.1.1 Computational fluid dynamics conditions

ANSYS CFX was employed for simulating the foldable aortic axial-flow blood pump. After considering both efficiency and accuracy, we adopted the RANS model to achieve faster convergence, and utilized the SST k- ω model as the turbulence model for more accurate prediction of flow onset and separation phenomena within the flow field. Blood was modeled as a viscous incompressible Newtonian fluid with viscosity 0.0035 Pa. s and density 1055 kg. m ^-3^. The inlet boundary condition was set as mass flow rate 0.087917 kg. s ^-3^ (equivalent to volumetric flow rate 5L. min ^-1^), with outlet pressure fixed at 100 mmHg, which is the recommended average blood pressure for Wang, (2025).

For the cage-free pump simulation, the impeller domain rotated at 8,000 rpm. The inlet and outlet interfaces connected to guide vane domains used ‘Frozen Rotor’ treatment, while other interfaces used stationary-stationary connections. All walls applied no-slip condition.

For the caged pump simulation, the impeller domain was fully enclosed by guide vanes and cage filaments, with all surrounding surfaces using rotor-stator interfaces. The SIMPLEC algorithm was selected for faster convergence, with residual criteria set to $10^{-5}$ .

#### 2.1.2 Mesh generation

Focusing on the expanded state flow analysis, the pump design featured maximum diameter 20 mm, length 57.5  mm, and hub radius 8 mm. The pump structure comprised inlet guide vanes, impeller, outlet guide vanes, and a cage housing.

To investigate the influence of cage filaments on the performance of an axial flow blood pump, this study conducted simulations for both filament-free and filament-equipped pump configurations. To minimize the impact of inlet/outlet boundary conditions in calculation on the physical flow characteristics while ensuring uniform inlet flow and fully developed outlet flow, extended segments were incorporated at both the inlet and outlet of the axial flow blood pump, thereby enhancing simulation accuracy.

Although the three-dimensional computational domain should ideally replicate the actual blood flow field precisely, the extremely narrow gaps between pump components and the acute-angle regions formed between cage filaments and vascular walls presented significant modeling challenges. These geometric features would severely compromise mesh quality and impede simulation convergence. Therefore, appropriate simplifications were implemented during 3D flow domain modeling, with the vascular wall in the caged pump configuration set to intersect the cage filaments at their mid-cross-section.

The computational domain was discretized using ICEM software. Unstructured polyhedral meshes were generated for the fluid domains of both pump variants, with interface surfaces connecting adjacent computational subdomains. Despite geometric simplifications, the 3D flow domain retained minute clearances. To better resolve boundary layer development on blade surfaces and blood flow patterns around cage filaments within tip clearance regions, localized mesh refinement was applied to select blade walls and cage filament surfaces. A grid independence study was performed to optimize the balance between computational efficiency and accuracy, ultimately determining that a mesh count of 3.8 million elements delivered stable and precise results.

#### 2.1.3 Hemolysis calculation

Currently, most quantitative studies on hemolysis are based on the power-law model proposed by Giersiepen et al. (1990) in 1990, expressed as:

$$\text{HI }\left(\%\right)=\frac{\Delta \text{Hb}}{\text{Hb}}=C\tau^{\alpha }t^{\beta }$$ (1)

where HI represents the hemolysis index, defined as the ratio of increased plasma free hemoglobin concentration to total plasma free hemoglobin concentration. A higher value indicates greater hemolysis severity. τ denotes the shear stress acting on red blood cells (unit: Pa), t is the exposure time (unit: s), Cα and β are empirical constants with values C=3.62,α=2.416,β=0.785 Grigioni et al. (2005). This model establishes the critical relationship between shear stress, its duration of action, and hemolysis.

Equation 1 demonstrates that calculating shear stress magnitude is essential for determining the hemolysis index. Given the complex three-dimensional flow field within axial flow blood pumps, the tensor form of shear stress must be converted to a scalar form for substitution into the power-law model. The tensor expression of shear stress is:

$$\tau_{ij}=\mu \frac{\partial v_{i}}{\partial x_{j}}$$ (2)

where represents blood viscosity.

Both viscous shear stress and Reynolds shear stress in turbulent flow contribute to erythrocyte damage Yen et al. (2014). The total stress tensor can be expressed as the sum of viscous and Reynolds stresses Mitoh et al. (2020):

$$\tau_{ij}=\sigma_{ij}+\left(-pv_{i}v_{j}\right)$$ (3)

In Equation 3:

$$\sigma_{ij}=\mu \left(\frac{\partial v_{i}}{\partial x_{j}}+\frac{\partial v_{j}}{\partial x_{i}}\right)$$ (4)

$$pv_{i}v_{j}=\left(\begin{array}{ccc} pv_{1}^{2} & pv_{1}v_{2} & pv_{1}v_{3}\\ pv_{2}v_{1} & pv_{2}^{2} & pv_{2}v_{3}\\ pv_{3}v_{1} & pv_{3}v_{2} & pv_{3}^{2} \end{array}\right)$$ (5)

Blood is treated as an incompressible Newtonian fluid within the pump, where $\sigma_{ij}$ denotes viscous shear stress, μ is the density constant, $v_{1},v_{2},v_{3}$ , denote velocity components along the x-, y-, and z-axes, respectively. Finally, the scalar shear stress expression derived from Bludszuweit’s method Bludszuweit (1995) is:

$$\tau ={\left[\frac{1}{6}\underset{i,j}{\sum }\left(\tau_{ij}-\tau_{jj}\right)+\underset{i,j}{\sum }\tau_{ij}^{2}\right]}^{1/2}$$ (6)

Based on the above principles, this study employs the Lagrangian method to track erythrocyte trajectories and calculate cumulative damage to obtain HI. The initial damage to red blood cells is assumed to be 0, and the blood damage $d_{p,i}$ experienced by a single erythrocyte during time step is given by:

$$d_{p,i}=362\cdot \tau_{i-1}^{2416}\cdot \Delta t_{i}^{0785}$$ (7)

The cumulative damage along the trajectory for a single erythrocyte is calculated as:

$$D_{p,i}=D_{p,i-1}+\left(1-D_{p,i-1}\right)\times d_{p,i}$$ (8)

where p denotes the trajectory index, and the initial damage $D_{0}$ is 0. The average damage HI experienced by erythrocytes passing through the blood pump, which serves as HI for evaluating the hemolytic performance of the axial flow blood pump, is obtained by averaging the cumulative results:

$$\text{HI}=\frac{1}{N}\overset{N}{\underset{i=1}{\sum }}D_{i}$$ (9)

The Lagrangian method for hemolysis prediction requires calculating the cumulative damage experienced by individual erythrocytes along their trajectories within the blood pump. The final hemolysis result is obtained by averaging the damage levels across all trajectories. To verify the independence of the calculated hemolysis results from the number of trajectories, this study conducted a relevant analysis using the initial blood pump model. The analysis involved hemolysis calculations with trajectory numbers ranging from 50 to 1,035, ultimately determining that 500 trajectories provided optimal efficiency and accuracy. Table 1 shows the results of path line independence verification.

**TABLE 1:** Path line independence verification.

No.	Number of path lines	Hemolysis value ( $\times 10^{-2}$ %)
1	50	1.5071
2	100	1.2224
3	200	1.3349
4	300	1.3717
5	400	1.3021
6	500	1.3162
7	700	1.3203
8	1,035	1.3151

### 2.2 Response surface methodology

RSM, which combines mathematical and statistical approaches, is particularly valuable for optimizing responses influenced by multiple variables in structural optimization problems (Kang et al., 2010; Park, 2010). Common RSM designs include Central Composite Design (CCD) and Box-Behnken Design (BBD). As BBD proves more economical and efficient for problems with 3-5 factors, this study adopts BBD for subsequent response surface optimization analysis. Plackett-Burman (PB) design is primarily employed when dealing with numerous factors where the significance of their effects on responses remains uncertain. This method effectively identifies influential factors with minimal experimental runs Zhou et al. (2011), and thus serves as the basis for screening significant factors in this study.

Using head and HI as response values, this section conducts significance analysis and screening of key structural parameters for the axial flow blood pump through PB design. The optimization objectives are achieving higher head while minimizing hemolysis under high-head conditions. Seven parameters are selected for significance analysis: impeller inlet angle $\theta_{1}$ , impeller outlet angle $\theta_{2}$ , impeller wrap angle $\theta_{3}$ , diffuser inlet angle $\theta_{4}$ , diffuser wrap angle $\theta_{5}$ , Front-Guide-Vane-Impeller Gap (F-I Gap) $L_{1}$ , and Impeller-Diffuser Gap $L_{2}$ .

Each structural parameter is assigned upper and lower critical bounds, designated as high level (+1) and low level (−1) respectively, as detailed in Table 2. For k=N−1 factor variables, PB design requires the number of runs N to be a multiple of 4 (i.e., N=12,20,… ). To estimate error, the design should include 1-3 dummy variables, making the actual number of variables fewer than N–1. This study employs a 12-run PB design, with numerical simulations performed for each set of structural parameters to obtain head and HI values. The most significant five factors affecting these responses are selected for subsequent response surface optimization analysis.

**TABLE 2:** Experimental design factors and levels.

No.	Factor	Low level (−1)	High level (1)
A	Impeller inlet angle	12	22
B	Impeller outlet angle	53	73
C	Impeller wrap angle	150	250
D	Diffuser inlet angle	15	35
E	Diffuser wrap angle	100	160
F	F-I gap	1.5	3.5
G	Impeller-diffuser gap	1.5	3.5

The significant factors - impeller inlet angle $\theta_{1}$ , impeller outlet angle $\theta_{2}$ , diffuser inlet angle $\theta_{4}$ , diffuser wrap angle $\theta_{5}$ , and Impeller-Diffuser Gap $L_{2}$ are treated as independent variables. These parameters are assigned three levels (high: +1, medium: 0, low: –1) based on previously determined ranges, with the medium level representing the arithmetic mean of high and low levels, for BBD implementation.

Using Design-Expert 13 software, a 5-factor 3-level BBD scheme is generated. Numerical simulations corresponding to this scheme yield respective head and HI values. Regression analysis performed with Design-Expert 13 produces multivariate quadratic regression prediction models for head and HI, ultimately leading to optimized structural parameters.

## 3 Results and discussion

### 3.1 Response surface optimization design

#### 3.1.1 Plackett-Burman experimental design

According to the settings in Section 2.1, numerical simulations were performed on the initial model, yielding a simulated head value of 2.1955 m and a simulated HI value of $1.3162\times 10^{-2}\;\%$ .

**FIGURE 3:**
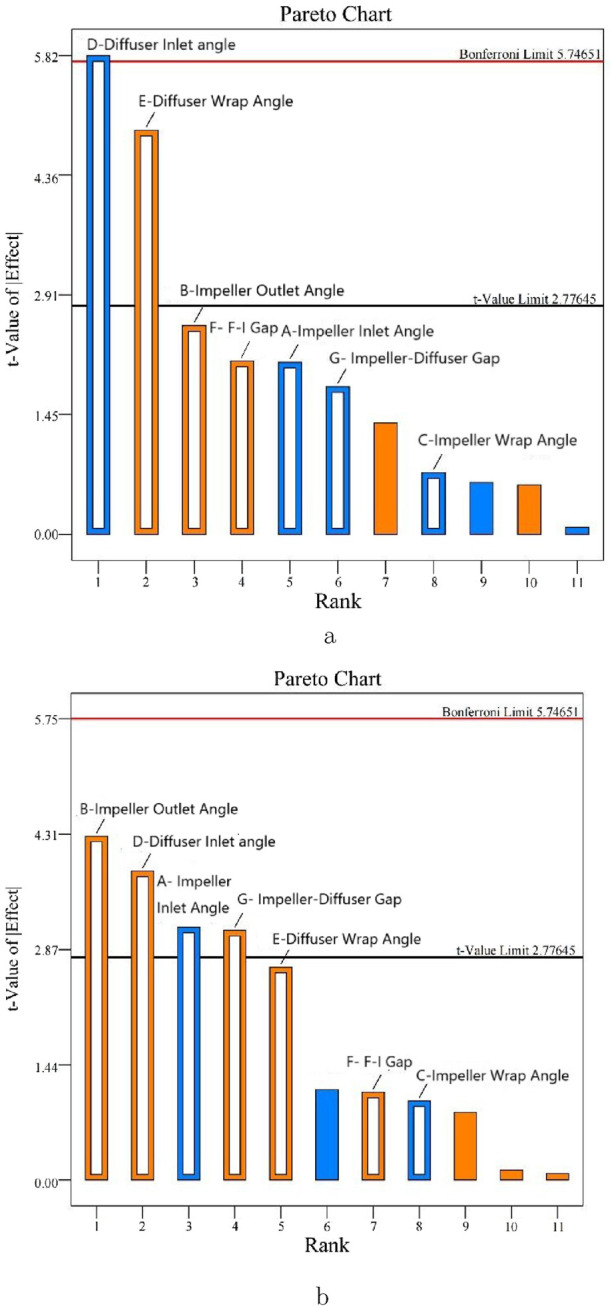
Pareto charts showing the effects of **(a)** impeller geometry on head and **(b)** clearance on hemolysis index.

Through BBD, 12 sets of simulation experiments were designed for significance analysis of the 7 factors. Figure 3 shows the Pareto charts of these 7 factors’ effects on the response values, where Figure 3a has head as the response variable and Figure 3b has HI as the response variable.

From Figure 3a, it can be seen that factor D and factor E have significant effects on head. From Figure 3b, factors A , B , D , and G show significant effects on HI. Multiple regression fitting was performed on the experimental design and simulation data to obtain coded regression equations for head $\left(Y_{1}\right)$ and HI $\left(Y_{2}\right)$ , as shown in Equations 10, 11 respectively. Combined with the Pareto charts, factors B , E , and F have positive effects on head, while factors A , C , D , and G have negative effects. For HI, factors B , D , E , F , and G show positive effects, while factors A and C have negative effects.

The corresponding multivariate quadratic regression prediction models were obtained. Equations 10, 11 represent the regression equations for head and HI respectively:

$$\begin{array}{ll} & Y_{1}=1.84-0.0514A+0.0624B-0.0184C-0.1432D+0.1209E\\ & \;+0.0518F-0.0441G \end{array}$$ (10)

$$\begin{array}{ll} & Y_{2}=1.13-0.0376A+0.0511B-0.01118C+0.0459D+0.0316E\\ & \;+0.0131F+0.0371G \end{array}$$ (11)

From the constructed regression equations, it can be observed that the P-values for factors D and E are both less than 0.01, indicating that diffuser inlet angle D and diffuser wrap angle E have highly significant effects on head, with the significance order being D>E . For HI, factors A , B , D , and G all have P-values less than 0.05, meaning impeller inlet angle A , impeller outlet angle B , diffuser inlet angle D , and Impeller-Diffuser Gap G significantly affect HI, with the significance order being B>D>A>G . Through analysis of variance (ANOVA), both models show P-values less than 0.05. This section selects the union of significant factors from both regression models, namely, impeller inlet angle A , impeller outlet angle B , diffuser inlet angle D , diffuser wrap angle E , and Impeller-Diffuser Gap G , as the research factors for subsequent response surface experiments.

#### 3.1.2 Response surface optimization and regression model construction

Numerical simulations were conducted according to the experimental design from Section 2.2, yielding 46 sets of head and HI data. Six center-point trials (numbers 2, 3, 31, 36, 38, and 44) were included to estimate experimental error. After regression analysis, the multivariate quadratic regression prediction model for pump head was obtained as follows:

$$\begin{array}{cc} Y_{3}= & \;2.18-0.0545A+0.0751B-0.087D+0.1692E+0.0861G\\ & -0.0302AB-0.0094AD-0.0027AE-0.0042AG+0.0264DE\\ & +0.0011BD+0.0027BE+0.0351BG-0.0294DG+0.0011EG\\ & -0.0498A^{2}-0.0274B^{2}-0.1434D^{2}-0.1085E^{2}-0.0932G^{2} \end{array}$$ (12)

To validate the model, ANOVA was performed on the mathematical model of pump head. The results showed the regression model was significant with P<0.0001 . The lack-of-fit term has a P=0.2005>0.05 ,which indicates that the test value of the head has a relatively high degree of fit with the predicted value. Factors A , B , D , E and G were all significant with P<0.05 , while interaction terms AB , AD , etc. Were insignificant with P>0.05 ,showing that the interaction between the structural parameters is not obvious. The quadratic terms $A^{2}$ , $B^{2}$ , $D^{2}$ , $E^{2}$ , and $G^{2}$ were all significant with P<0.05 . The model’s *R* ^2^ value of 0.9226 was close to 1, and the difference between adjusted *R* ^2^ $\left(R_{\mathrm{adj}}^{2}\right)$ and predicted *R* ^2^ $\left(R_{\mathrm{pred}}^{2}\right)$ was less than 0.2, suggesting that the relationship between each factor and the head is not a simple linear one. The adequate precision was 16.2812, which demonstrates good fit and reliability.

From the ANOVA results, only two interaction terms with smallest P-values ( BG and AB ) were selected for further analysis. Using Equation 12, response surface and contour plots were generated in Design-Expert 13.

**FIGURE 4:**
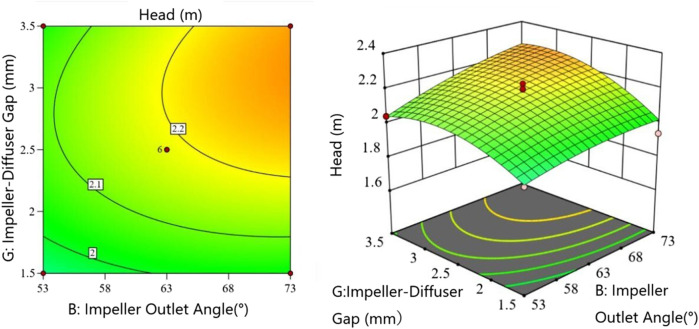
Interaction between impeller outlet angle and impeller-diffuser gap.

Figure 4 shows the interaction between impeller outlet angle (B) and impeller-diffuser gap (G) with other factors at 0 level (all subsequent interaction analyses maintain this condition). The head increases with both parameters, but the rate of increase slows when the impeller-diffuser gap exceeds 2.5 mm. The contour lines (denser on the right) indicate that the impeller-diffuser gap has slightly greater influence on head than the impeller outlet angle within the tested range.

**FIGURE 5:**
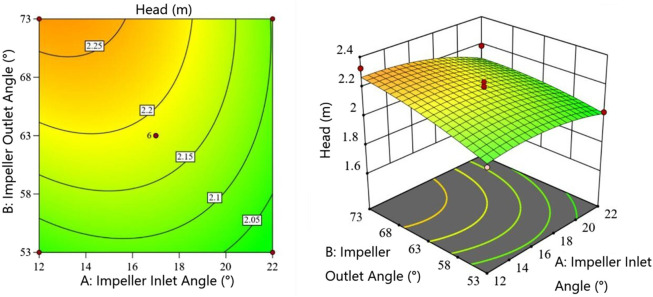
Interaction between impeller inlet angle and impeller outlet angle.

Figure 5 demonstrates the interaction between impeller inlet angle (A) and impeller outlet angle (B) . The pump head increases with decreasing impeller inlet angle and increasing outlet angle, with diminishing returns when the inlet angle falls below 18 ◦ and the outlet angle exceeds 63 ◦ .

Similarly, the multivariate quadratic regression model for HI was obtained:

$$\begin{array}{cc} Y_{4}= & \;1.31-0.0381A+0.0402B+0.0026D+0.0364E+0.0859G\\ & -0.0123AB-0.017AD-0.0531AE-0.0505AG+0.0437BD\\ & -0.0177BE+0.0418BG+0.0603DE+0.0207DG+0.0396EG\\ & -0.0665A^{2}-0.054B^{2}-0.0712D^{2}-0.0643E^{2}-0.0496G^{2} \end{array}$$ (13)

To validate the hemolysis prediction model, ANOVA was performed on the mathematical model of blood pump hemolysis. The results demonstrate that the regression model is highly significant (P<0.0001) with insignificant lack-of-fit (P=0.1295>0.05) . The main effects of factors A (Impeller inlet angle), B (Impeller outlet angle), E (Diffuser wrap angle), and G (Impeller-Diffuser Gap) were all significant (P<0.05) . Significant interaction effects (P<0.05) were observed for AE , AG , BD , BG , DE , and EG . Notably, while the Diffuser inlet angle D showed no significant individual effect (P>0.05) , its interactions with factors A and E were significant, which aligns with engineering experience. The significance of quadratic terms ( $A^{2}$ and $D^{2}$ , P<0.05 ) confirmed nonlinear relationships between parameters and HI.

The hemolysis prediction model exhibited excellent fitting performance: the multiple correlation coefficient $R^{2}=0.932$ approaches the ideal value of 1, the difference between adjusted $R_{\text{adj}}^{2}$ and predicted $R_{\text{pred}}^{2}$ coefficients is less than 0.2, and the signal-to-noise ratio (SNR) is significantly greater than 4, meeting all required metrics.

Compared with the head model, HI is influenced by more quadratic terms, ordered by significance as DE>AE>AG>BD>BG>EG . This indicates greater complexity in how axial flow blood pump structural parameters affect HI: parameters not only influence hemolysis performance independently but also alter flow field shear stress (τ) and red blood cell exposure time (t) through interactions, ultimately affecting HI. Accordingly, this study employed the same methodology as the head analysis, generating contour plots and response surfaces for these six significant interaction terms.

**FIGURE 6:**
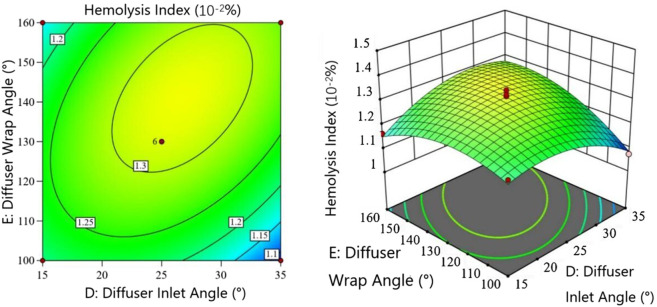
Interaction between Diffuser inlet angle D and Diffuser wrap angle E in the hemolysis model.

Figure 6 shows the interaction between diffuser inlet angle D and diffuser wrap angle E . The HI initially increases then decreases with increasing Diffuser inlet angle. When the Diffuser wrap angle is <110° , HI changes gradually with increasing Diffuser inlet angle until reaching 25 ◦ , after which it decreases rapidly to a minimum. The Diffuser wrap angle shows a similar trend, with HI first increasing then decreasing. The elliptical contour lines clearly demonstrate significant interaction effects. The Diffuser inlet and wrap angles determine the diffuser shape, which guides flow, increases pressure, and reduces circulation in blood pumps,so the interaction between them can greatly influence HI- results consistent with empirical knowledge.

**FIGURE 7:**
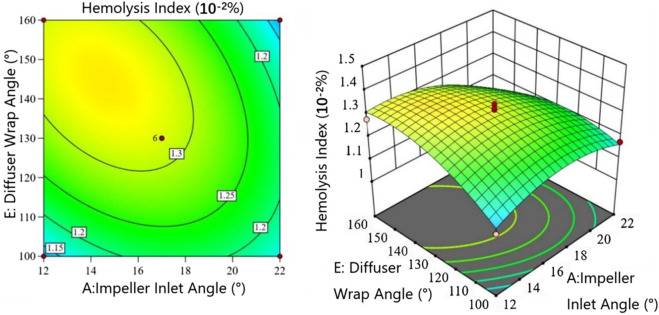
Interaction between umpeller inlet angle A and diffuser wrap angle E

Figure 7 presents the interaction between impeller inlet angle A and diffuser wrap angle E . When the Impeller inlet angle is small, HI changes sharply with increasing Diffuser wrap angle - first increasing rapidly, then stabilizing, before decreasing slightly. Maximum hemolysis occurs at Impeller inlet angles of 14 ◦ -16 ◦ and Diffuser wrap angles of 140 ◦ -150 ◦ . For Impeller inlet angles >18° , HI first increases then decreases with increasing Diffuser wrap angle, with less pronounced response surface variations. Although the Impeller inlet angle and Diffuser wrap angle independently affect their respective components, their interaction demonstrates the critical importance of impeller-diffuser matching on hemolysis. Proper matching reduces shear stress on erythrocytes in the flow path, while decreased turbulence and vortices shorten exposure times, consequently lowering HI.

**FIGURE 8:**
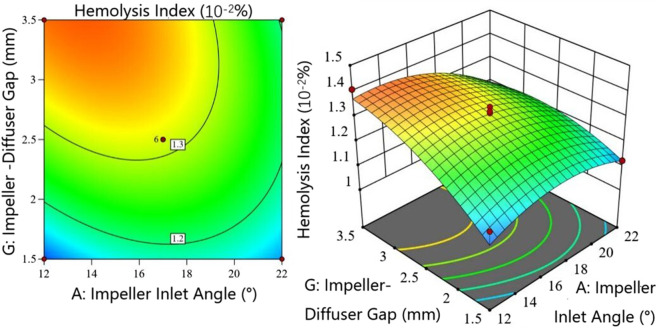
Interaction between impeller inlet angle and impeller-diffuser gap in the hemolysis model.

Figure 8 illustrates the interaction between impeller inlet angle A and impeller-diffuser gap G . When the Impeller inlet angle exceeds 20 ◦ , HI shows minimal variation with increasing impeller-diffuser gap, exhibiting a slight initial increase followed by a minor decrease. However, at smaller impeller inlet angles, HI increases monotonically with larger impeller-diffuser gap, demonstrating a steeper response surface. Contour analysis reveals that achieving equivalent HI reduction requires smaller adjustments to the impeller-diffuser gap compared to the Impeller inlet angle, indicating greater sensitivity to gap variations.

**FIGURE 9:**
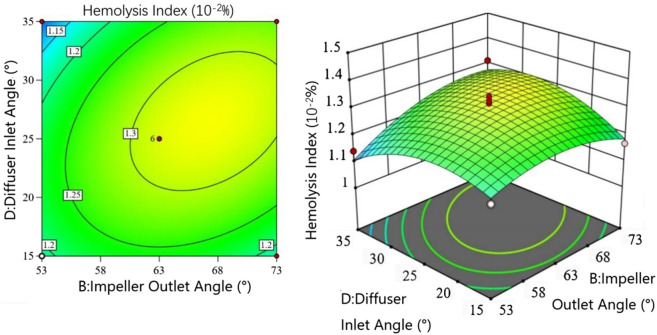
Interaction between impeller outlet angle and diffuser inlet angle in the hemolysis model.

Figure 9 presents the interaction between impeller outlet angle B and diffuser inlet angle D . When the Diffuser inlet angle approaches 15 ◦ , the impeller outlet angle has negligible effect on HI. At larger diffuser inlet angles, HI initially increases then decreases with simultaneous increases in both angles. Within certain ranges, increasing the impeller outlet angle enhances pump head but may elevate shear stress on erythrocytes, necessitating balanced optimization. The hydrodynamic alignment between impeller discharge flow and diffuser inlet channels critically determines HI levels, as misalignment induces turbulent shear stresses.

**FIGURE 10:**
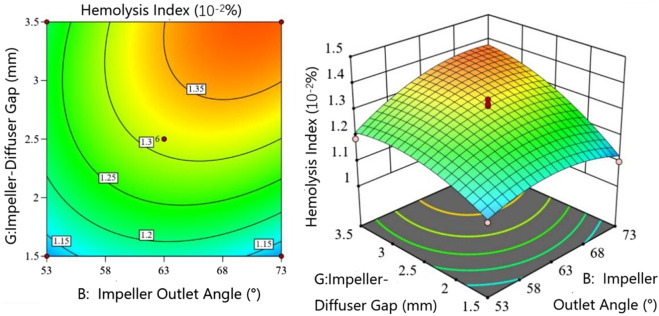
Interaction between Impeller outlet angle and Impeller-Diffuser Gap in the hemolysis model.

Figure 10 demonstrates the interaction between impeller outlet angle B and impeller-diffuser hap G . For gaps exceeding 3 mm, HI increases monotonically with impeller outlet angle. Below 3 mm, HI first rises then falls with increasing outlet angle, with diminished sensitivity at smaller gaps. This transition region represents a critical shear stress zone where flow transition quality between impeller and diffuser significantly impacts HI.

**FIGURE 11:**
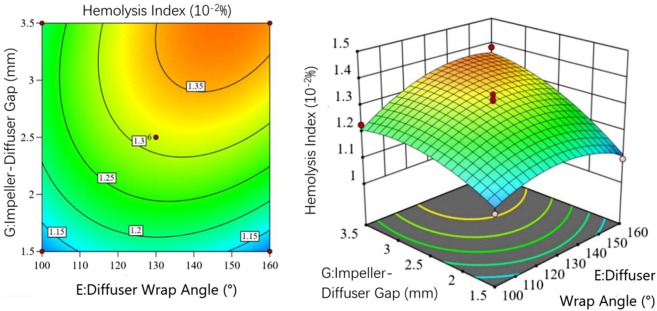
Interaction between Diffuser wrap angle and Impeller-Diffuser Gap in the hemolysis model.

Figure 11 examines the interaction between diffuser wrap angle E and impeller-diffuser gap G . For wrap angles below 140 ◦ , HI increases with simultaneous increases in both parameters. Beyond 140 ◦ , HI decreases with larger wrap angles at fixed gaps. Response surface gradients indicate heightened sensitivity to gap reductions below 2.5  mm, surpassing the influence of wrap angle variations.

The multi-objective optimization prioritized maximum head (weight = 2) over minimal HI (weight = 1) within design constraints. Using Equations 12, 13 with manufacturing tolerances, the optimal configuration was implemented and simulated under identical conditions to the center point. Table 3 compares performance metrics between initial and optimized designs.

**TABLE 3:** Comparison of initial and optimized pump parameters.

Model	$\theta_{1}$ /°	$\theta_{2}$ /°	$\theta_{4}$ /°	$\theta_{5}$ /°	$L_{2}$ /mm	Head/m	HI/ $10^{-2}$ %
Initial	17	63	25	130	2.5	2.1955	1.3162
Optimized	21	72	16	160	3.1	2.3460	1.0811

### 3.2 Influence and optimization of blade tip clearance on blood pump performance

**FIGURE 12:**
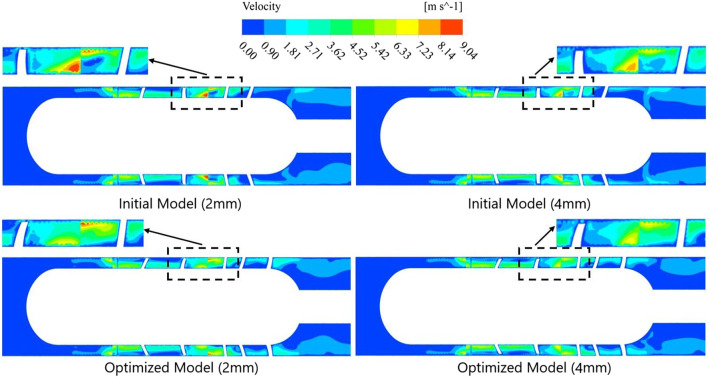
Contour plots of velocity distribution for initial and optimized models before and after increasing blade tip clearance.

In addition to the structural parameters studied in Section 3.1, blade tip clearance is another important factor affecting the performance of axial flow pumps, which will be studied separately here. Through numerical simulation, the flow field distribution and pathline trajectories of the blood pump before and after optimization were obtained. Figure 12 shows the velocity distribution contours of the initial and optimized models under different blade tip clearances, where the clearances are set to 0.2 mm and 0.4  mm, respectively.

From the figure, it can be observed that the overall velocity variation patterns of all models are similar. The blood flow remains relatively stable in the front guide vane section. As it enters the impeller, the blood receives both circumferential and axial acceleration due to the gradual increase in the impeller blade angle from inlet to outlet and the high-speed rotation, resulting in a significant velocity gradient in the impeller section. Particularly at the interface between the impeller and the rear guide vane, all four models exhibit small high-speed regions.

However, comparisons reveal that the optimized models with the same tip clearance have smaller high-speed regions than the initial models, with smoother velocity transitions. This indicates that the optimized models provide better control over the blood flow as it moves from the impeller trailing edge to the diffuser leading edge, thereby improving hydraulic performance. Furthermore, for the same model, the 0.4 mm clearance case shows a smaller high-speed region compared to the 0.2 mm case. This is because a larger tip clearance reduces the effective working area of the impeller blades, leading to increased leakage flow and corresponding decreases in both velocity gradient and head. Consequently, the risk of red blood cell damage is significantly reduced.

**FIGURE 13:**
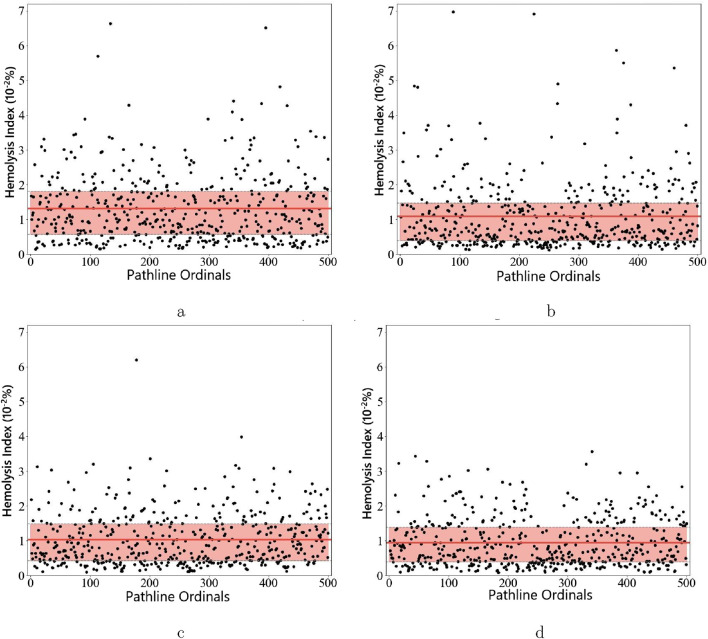
Scatter plots of path line hemolysis value distribution for initial and optimized models before and after increasing blade tip clearance. **(a)** Initial Model (0.2 mm). **(b)** Initial Model (0.4 mm). **(c)** Optimized Model (0.2 mm). **(d)** Optimized Model (0.4 mm).

This study predicts the Hemolysis Index (HI) of the blood pump using the particle tracking method. For each pump model, 500 complete pathlines were randomly selected, and the HI for each pathline was calculated. The upper and lower quartiles were used to indicate the main distribution range of HI for each model. Figure 13 shows the resulting HI distribution plots, where the red solid lines represent the average HI for the four models.

The initial models with 0.2  mm and 0.4 mm clearances exhibit HI upper and lower quartile ranges of $\left(0.5631,1.8018\right)\times 10^{-2}$ % and $\left(0.4255,1.4757\right)\times 10^{-2}$ %, respectively. The optimized models with 0.2  mm and 0.4 mm clearances show ranges of $\left(0.3874,1.4689\right)\times 10^{-2}$ % and $\left(0.3815,1.3870\right)\times 10^{-2}$ %, respectively.

These results demonstrate that the optimized models have lower HI distribution ranges compared to the initial models under the same tip clearance. Additionally, for the same model, the smaller clearance case shows higher HI ranges than the larger clearance case. This indicates that the hemolysis performance of the pump is improved after response surface optimization. Given that the ideal mean blood pressure is up to 100 mmHg, and considering the patient’s residual cardiac function, along with the increase in head caused by the narrowed flow path due to the addition of cage filaments, we deemed a head of 1.0811 m acceptable. Furthermore, the reduced hemolysis value achieved by the 0.4 mm model is crucial for the patient. Therefore, although increasing the tip clearance from 0.2  mm to 0.4 mm may cause some hydraulic losses, it significantly reduces the risk of red blood cell damage, aligning better with the objectives of this study.

Regarding the simulation results, there is limited numerical research on foldable pumps, making it difficult to validate the correctness of our findings through comparisons. Current studies by Hirschhorn’s group Hirschhorn et al. (2020) and Bisirri. (2018) primarily focus on pressure and material strain, whereas this work emphasizes pump head and HI. Therefore, direct comparisons are challenging. Future work will further investigate the pressure and shear stress distributions around the pump to analyze its mechanical performance in depth and compare it with other relevant studies.

In PVAD-related research, Chen et al. (2024) developed a hemolysis prediction model for a self-designed PVAD, studying HI variations under steady and pulsatile flows. Xu et al. (2024) proposed a numerical method to evaluate hemolysis and thrombosis risks in PVADs, calculating the modified index of hemolysis. Both studies report HI values that are approximately four times higher than those of our optimized pump after conversion. This discrepancy may stem from differences in pump diameter. PVADs typically have radii smaller than 7 mm, with Chen’s PVAD having a radius of only 5.6 mm. For commercial PVADs, Roberts et al. (2020) studied the performance of Impeller CP, finding the modified hemolysis index of it to be 2.78 ± 0.69, which is much higher than the foldable blood pump. This results in significant differences in rotational speed, with PVAD simulations typically set at 20,000–30,000 rpm, compared to 8,000 rpm in this study. Consequently, the shear stress near the pump may vary considerably, leading to differences in hemolysis performance and highlighting the advantages of foldable blood pumps. However, this may also suggest that our boundary conditions overestimated the influence of impeller working radius on rotational speed. Future studies will further explore the relationship between foldable pump impeller radius and its flow rate and rotational speed.

### 3.3 Limitations

This study is primarily a focused case study on a specific foldable pump geometry. While the developed optimization methodology demonstrates significant potential, the generalizability of the quantitative results to fundamentally different pump architectures has not been empirically validated and remains a subject for future research.

While the numerical models employed in this study offer valuable insights into blood damage trends, their limitations must be emphasized. The current numerical prediction of hemolysis is not fully reliable for absolute quantification, as models are based on simplified empirical laws. Consequently, the reported values should be used for controlled relative comparisons rather than as direct predictors of clinical outcomes. Furthermore, the Response Surface Methodology (RSM) is constrained by its underlying experimental design and may not capture all complex, non-linear interactions beyond the studied parameter space.

To robustly confirm these findings, key simulations must be validated through targeted *in vitro* experiments. Essential measurements include quantifying hemolysis via plasma-free hemoglobin (PFH) levels, assessing platelet activation and cellular morphology using flow cytometry or microscopy, and validating critical flow patterns with experimental techniques such as Particle Image Velocimetry (PIV). This combined approach of simulation and experimentation is crucial for translating computational trends into clinically relevant conclusions.

The RANS-based stress decomposition, while computationally efficient and well-suited for engineering analysis, presents certain limitations. The reliance on the eddy-viscosity hypothesis implies that the Reynolds stress is modeled as being isotropic and linearly related to the mean strain rate. This assumption may not fully capture the anisotropic nature of turbulence in highly complex, three-dimensional, or transient flows Faghih and Sharp (2019). Consequently, while the model is effective for identifying relative trends and areas of elevated stress risk, the absolute values of the predicted Reynolds stresses and any derived hemolysis indices should be interpreted with caution. Future work should include validation against experimental data, such as particle image velocimetry (PIV), to quantify the model’s predictive fidelity in the specific geometries considered.

The conclusions of this study are framed within the context of the chosen numerical models. A key consideration is the use of a scalar stress measure to predict hemolysis. While the Bludszuweit scalar shear stress model enabled a tractable and efficient optimization workflow, it is recognized that such formulations abstract the full stress tensor into a single value, which may not be fully consistent with the fundamental mechanisms of red blood cell damage under complex flow conditions Faghih and Keith Sharp (2016). Future work will involve employing more physiologically accurate, tensor-based blood damage models to further validate the findings presented here. However, the consistent relative performance trends observed during optimization suggest that the primary conclusions regarding design superiority are robust.

## 4 Conclusion

This study designed a foldable axial blood pump suitable for the physiological dimensions of the ascending aorta. Using response surface methodology, we screened for the most significant structural parameters and optimized the initial model. Through CFD simulations, we investigated the effects of blade tip clearance on the hydraulic and hemolytic performance of the blood pump. It is important to note that the findings of this study are specific to the pump design investigated herein and are not universally applicable.

The PB test identified five significant factors from seven candidate structural parameters: impeller inlet angle, impeller outlet angle, diffuser inlet angle, diffuser wrap angle, and impeller-diffuser gap. Using pump head and HI as response values, we established predictive models through BBD and analyzed significant interaction terms. The results showed that these factors primarily affect pump performance by influencing the matching relationship between the impeller blades and diffuser vanes.

The optimal structural parameters were determined as: impeller inlet angle 21° , impeller outlet angle 72° , diffuser inlet angle 16° , diffuser wrap angle 160° , and impeller-diffuser gap 3.1mm . The final optimized model achieved a head of 2.346m (6.9% improvement over the initial model) and HI of $1.0811\times 10^{-4}$ (17.9% reduction compared to the initial model).

Furthermore, we investigated the effect of blade tip clearance. While maintaining the overall pump diameter, we increased the tip clearance from 0.2  mm to 0.4 mm for both initial and optimized models. The optimized pump with 0.2 mm clearance serves as a reference for caged blood pumps, while the same model provides a prototype for subsequent cage filament studies.

Comparative performance analysis revealed that increased tip clearance significantly reduces pump head while decreasing high-velocity regions, thereby improving hemolytic performance. For the optimized pump, the HI quartile range decreased from $\left(0.3874,1.4689\right)\times 10^{-2}\%$ (0.2 mm) to $\left(0.3815,1.3870\right)\times 10^{-2}\%$ (0.4 mm) , with noticeable reductions in both maximum and minimum values. Notably, the optimized models outperformed their initial counterparts at both clearance levels. For comparison, the initial model showed HI quartile ranges of $\left(0.5631,1.8018\right)\times 10^{-2}\%$ and $\left(0.4255,1.4757\right)\times 10^{-2}\%$ for 0.2  mm and 0.4 mm clearances, respectively.

This study demonstrates that the influence mechanisms of significant factors on pump head and HI are complex and require case-specific parameter selection. While increased tip clearance reduces head, it correspondingly improves HI. Therefore, foldable blood pumps can appropriately increase tip clearance when sufficient head is maintained.

## Data Availability

The datasets presented in this study can be found in online repositories. The names of the repository/repositories and accession number(s) can be found in the article/Supplementary Material.
