# Supplementary figures and images for: Mechanically-foldable axial flow blood pump: response-surface-based structural optimization and hemolytic performance evaluation
Source: Front Physiol. 2025 Dec 18;16:1632333. doi: 10.3389/fphys.2025.1632333 (PMC12756083; doi:10.3389/fphys.2025.1632333)

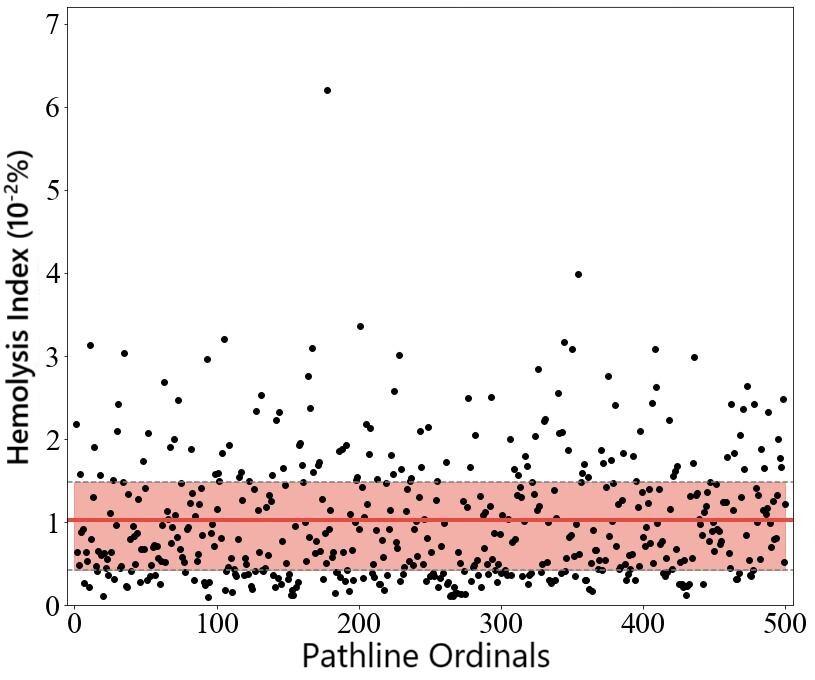

Supplement: Supplementary file 1 [file Image3.jpeg]

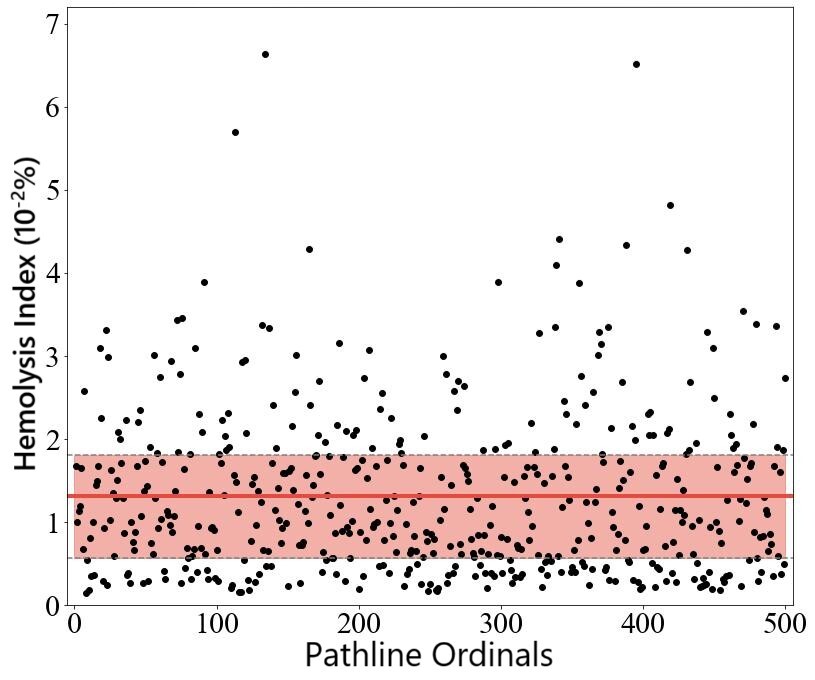

Supplement: Supplementary file 2 [file Image1.jpeg]

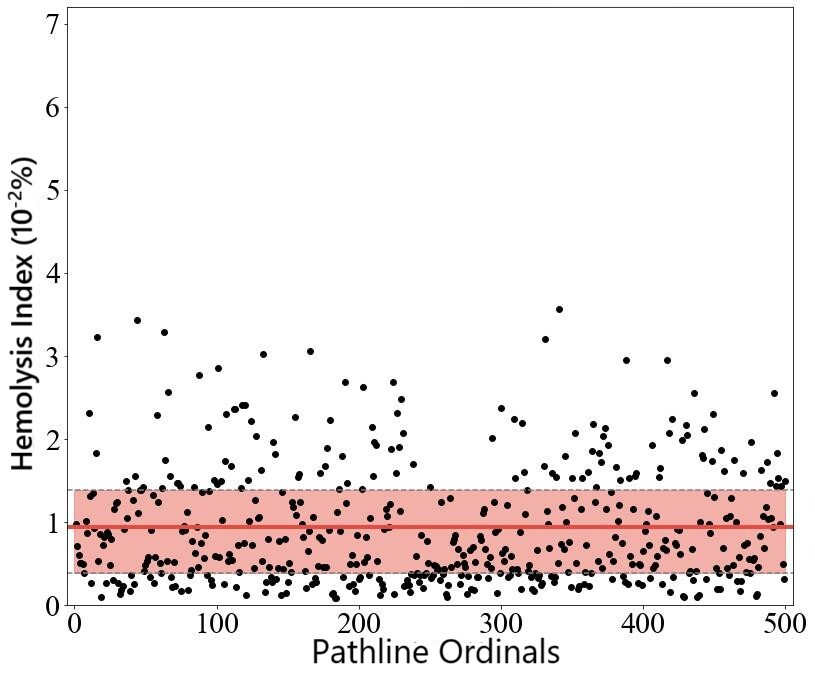

Supplement: Supplementary file 3 [file Image4.jpeg]

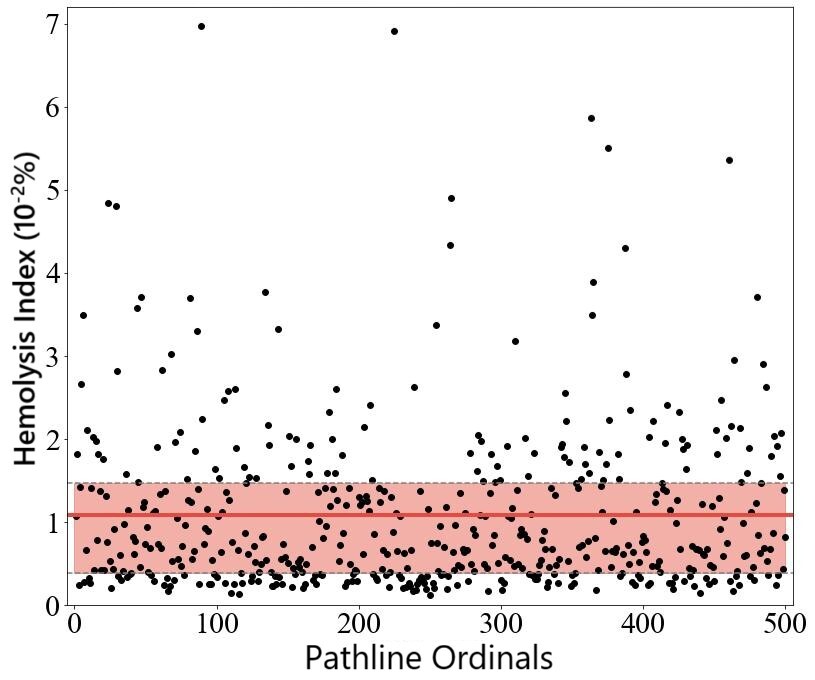

Supplement: Supplementary file 4 [file Image2.jpeg]
