# Supplementary material for: Mechanically-foldable axial flow blood pump: response-surface-based structural optimization and hemolytic performance evaluation
Source: Front Physiol. 2025 Dec 18;16:1632333. doi: 10.3389/fphys.2025.1632333 (PMC12756083; doi:10.3389/fphys.2025.1632333)
Supplement: Supplementary file 5 [file DataSheet1.pdf]

Supplementary Material

1 SUPPLEMENTARY DATA

Table S1. Grid independence verification

No.	Number of grids	Head (m)
1	1,300,350	1.9664
2	2,467,628	2.1980
3	3,164,460	2.2288
4	3,803,674	2.1955
5	5,141,930	2.1834
6	7,249,891	2.1961

Table S2. Path line independence verification

No.	Number of path lines	Hemolysis value ($\times 10^{-2}\%$)
1	50	1.5071
2	100	1.2224
3	200	1.3349
4	300	1.3717
5	400	1.3021
6	500	1.3162
7	700	1.3203
8	1035	1.3151

Table S3. Plackett-Burman design plan and results

No.	A	B	C	D	E	F	G	Head (m)	Hemolysis ($\times 10^{-2}\%$)
1	1	1	-1	1	1	-1	1	1.7753	1.2706
2	-1	1	1	-1	1	1	1	2.1372	1.2496
3	1	-1	1	1	-1	1	1	1.4669	1.1050
4	-1	1	-1	1	1	1	-1	2.1066	1.2531
5	-1	-1	1	-1	1	1	1	2.0467	1.0998
6	-1	-1	-1	1	-1	1	1	1.5737	1.1739
7	1	-1	-1	-1	1	1	-1	2.0401	1.0511
8	1	1	-1	-1	-1	-1	1	1.7696	1.0801
9	1	1	1	-1	-1	-1	1	2.0204	1.0020
10	-1	1	1	1	-1	-1	-1	1.5991	1.2074
11	1	-1	1	1	1	-1	-1	1.6533	1.0218
12	-1	-1	-1	-1	-1	-1	-1	1.8791	0.9981

Table S4. The significance analysis results of various factors of head

Source	Sum of Squares	df	Mean Square	*F* Value	*P* Value
Model 1	0.5593	7	0.0799	11	0.0177
A	0.0317	1	0.0317	4.36	0.1049
B	0.0467	1	0.0467	6.42	0.0643
C	0.0041	1	0.0041	0.5592	0.4961
D	0.246	1	0.246	33.86	0.0043
E	0.1753	1	0.1753	24.13	0.008
F	0.0322	1	0.0322	4.44	0.103
G	0.0233	1	0.0233	3.21	0.1475

Table S5. The significance analysis results of various factors of hemolysis value

Source	Sum of Squares	df	Mean Square	*F* Value	*P* Value
Model 2	0.1059	7	0.0151	8.85	0.0261
A	0.017	1	0.017	9.93	0.0345
B	0.0313	1	0.0313	18.33	0.0128
C	0.0017	1	0.0017	0.9737	0.3796
D	0.0253	1	0.0253	14.81	0.0183
E	0.012	1	0.012	7.02	0.057
F	0.0021	1	0.0021	1.2	0.3347
G	0.0165	1	0.0165	9.68	0.0358

Table S6. Box-Behnken design plan and results

No.	A	B	D	E	G	Head (m)	Hemolysis ($\times 10^{-2}\%$)
1	-1	1	0	0	0	2.3257	1.2763
2	0	0	0	0	0	2.2288	1.3040
3	0	0	0	0	0	2.0996	1.2816
4	0	-1	0	0	1	2.0424	1.1888
5	-1	0	0	0	-1	2.0096	1.1513
6	0	0	0	-1	-1	1.7269	1.0961
7	1	0	0	0	-1	1.8966	1.1236
8	0	-1	0	-1	0	1.7963	1.0905
9	0	0	1	-1	0	1.6724	1.0723
10	0	0	-1	1	0	2.1449	1.1639
11	1	-1	0	0	0	2.0165	1.1448
12	-1	-1	0	0	0	2.0330	1.1913
13	0	0	0	1	-1	2.0414	1.0924
14	0	-1	1	0	0	1.8995	1.1410
15	0	0	0	1	1	2.1956	1.3829
16	0	0	-1	0	1	2.2299	1.2368
17	0	0	0	-1	1	1.8768	1.2282
18	0	1	0	-1	0	1.8910	1.2352
19	0	0	1	0	-1	1.8319	1.1149
20	0	1	0	0	-1	1.9373	1.0995
21	0	1	-1	0	0	2.1135	1.1652
22	1	0	0	0	1	2.0807	1.1787
23	0	1	0	1	0	2.3721	1.2811
24	-1	0	0	-1	0	1.9151	1.1073
25	0	1	1	0	0	2.0419	1.3118
26	0	-1	0	1	0	2.1694	1.2071
27	0	0	-1	-1	0	1.9450	1.2146
28	1	1	0	0	0	2.1883	1.1807
29	1	0	-1	0	0	1.9722	1.1753
30	-1	0	0	0	1	2.2104	1.4084
31	0	0	0	0	0	2.1834	1.3117
32	1	0	0	-1	0	1.7977	1.1720
33	-1	0	-1	0	0	2.0686	1.2046
34	-1	0	0	1	0	2.2774	1.2782
35	1	0	0	1	0	2.1492	1.1305
36	0	0	0	0	0	2.1961	1.3414
37	-1	0	1	0	0	1.9234	1.1812
38	0	0	0	0	0	2.1980	1.3234
39	0	0	1	0	1	1.9326	1.3187
40	0	0	1	1	0	1.9778	1.2628
41	0	-1	0	0	-1	1.9279	1.1151
42	1	0	1	0	0	1.7894	1.0841
43	0	1	0	0	1	2.1922	1.3406
44	0	0	0	0	0	2.1955	1.3162
45	0	-1	-1	0	0	1.9756	1.1691
46	0	0	-1	0	-1	2.0116	1.1157

Table S7. Analysis of variance results of head prediction model

Source	Sum of Squares	df	Mean Square	F Value	P Value
Model 3	1.1	20	0.0552	14.9	< 0.0001
A	0.0476	1	0.0476	12.83	0.0014
B	0.0902	1	0.0902	24.33	< 0.0001
D	0.1212	1	0.1212	32.68	< 0.0001
E	0.4579	1	0.4579	123.48	< 0.0001
G	0.1186	1	0.1186	31.98	< 0.0001
AB	0.0037	1	0.0037	0.9855	0.3303
AD	0.0004	1	0.0004	0.0953	0.7601
AE	0	1	0	0.0079	0.93
AG	0.0001	1	0.0001	0.0188	0.892
BD	5.06E-06	1	5.06E-06	0.0014	0.9708
BE	0.0029	1	0.0029	0.7865	0.3836
BG	0.0049	1	0.0049	1.33	0.2599
DE	0.0028	1	0.0028	0.7505	0.3946
DG	0.0035	1	0.0035	0.9325	0.3435
EG	4.62E-06	1	4.62E-06	0.0012	0.9721
A^2	0.0216	1	0.0216	5.84	0.0233
B^2	0.0065	1	0.0065	1.77	0.1958
D^2	0.1795	1	0.1795	48.42	< 0.0001
E^2	0.1028	1	0.1028	27.72	< 0.0001
G^2	0.0743	1	0.0743	20.05	0.0001
Residual	0.0927	25	0.0037		
Lack of Fit	0.0831	20	0.0042	2.16	0.2005
Pure Error	0.0096	5	0.0019		
Total	1.2	45			
R^2	0.9226		R^2_{adj}	0.8607	
R^2_{pred}	0.7109		Signal-to-Noise	16.2812	
